# The Effects of One-Point Mutation on the New Delhi Metallo Beta-Lactamase-1 Resistance toward Carbapenem Antibiotics and β-Lactamase Inhibitors: An In Silico Systematic Approach

**DOI:** 10.3390/ijms232416083

**Published:** 2022-12-16

**Authors:** Van-Thanh Tran, Viet-Hung Tran, Dac-Nhan Nguyen, Tran-Giang-Son Do, Thanh-Phuong Vo, Thi-Thao-Nhung Nguyen, Phuong Nguyen Hoai Huynh, Khac-Minh Thai

**Affiliations:** 1 Faculty of Pharmacy, University of Medicine and Pharmacy at Ho Chi Minh City, Ho Chi Minh City 700000, Vietnam; 2 Institute of Drug Quality Control Ho Chi Minh City, Ho Chi Minh City 700000, Vietnam

**Keywords:** New Delhi Metallo beta-lactamase-1 enzyme, alternative one-point mutation, in silico, molecular docking, molecular dynamics simulation

## Abstract

Antibiotic resistance has been becoming more and more critical due to bacteria’s evolving hydrolysis enzymes. The NDM-1 enzyme could hydrolyze not only carbapenems but also most of β-lactam’s antibiotics and inhibitors. In fact, variant strains could impose a high impact on the resistance of bacteria producing NDM-1. Although previous studies showed the effect of some variants toward antibiotics and inhibitors binding, there has been no research systematically evaluating the effects of alternative one-point mutations on the hydrolysis capacity of NDM-1. This study aims to identify which mutants could increase or decrease the effectiveness of antibiotics and β-lactamase inhibitors toward bacteria. Firstly, 35 different variants with a high probability of emergence based on the PAM-1 matrix were constructed and then docked with 5 ligands, namely d-captopril, l-captopril, thiorphan, imipenem, and meropenem. The selected complexes underwent molecular dynamics simulation and free energy binding estimation, with the results showing that the substitutions at residues 122 and 124 most influenced the binding ability of NDM-1 toward inhibitors and antibiotics. The H122R mutant decreases the binding ability between d-captopril and NDM-1 and diminishes the effectiveness of this antibiotic toward Enterobacteriaceae. However, the H122R mutant has a contrary impact on thiorphan, which should be tested in vitro and in vivo in further experiments.

## 1. Introduction

**Figure 1:**
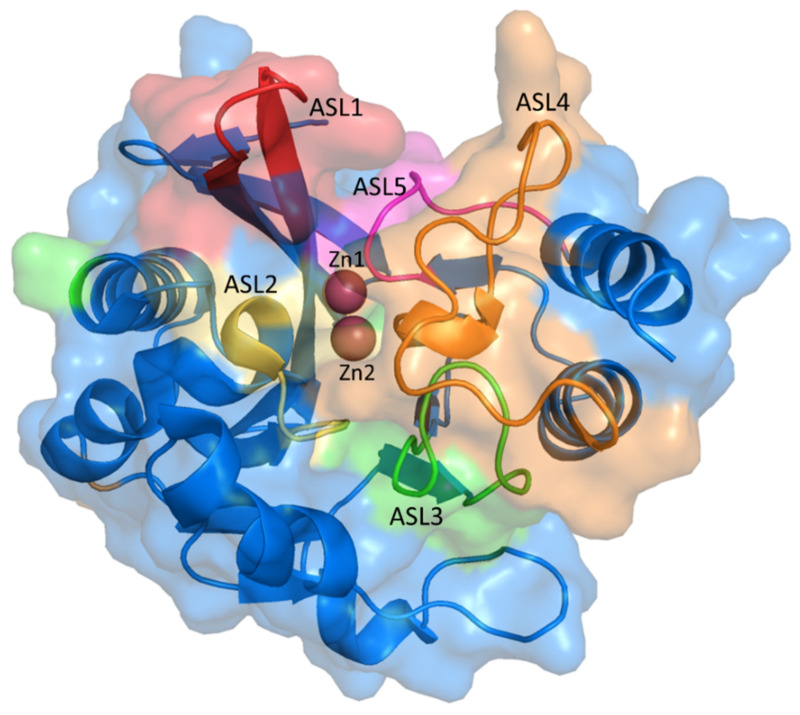
The 3D structure of NDM-1 (PDB ID: 5ZJ2) with the active site loops (ASL).

New Delhi-Metallo-beta-lactamase-1 (NDM-1) is an enzyme that causes severe drug resistance, which is essentially found in Enterobacteriaceae with the role of hydrolyzing carbapenem. The NDM-1 enzyme consists of 270 residues (27.5 kDa) belonging to the B1 Class of Metallo-β-lactamase (MBL) with two zinc ions in the active site. While the coordination of the carbonyl oxygen atom in the ß-lactam ring with Zn1 could help the nucleophile directly attack hydroxide ions in the active site, the coordination of Zn2 with N-amides of the substrate and the oxygen carboxylate of the adjacent ring induces the protonation of N atom and the cleavage of the CN bonds. This mechanism would result in the opening of the ß-lactam ring and prohibit antibiotic activity [1,2]. The 3D structure of the NDM-1 protein is shown in Figure 1.

NDM-1′s sequence is similar to other MBLs, with the active form including dimer and monomer conformations. The monomer structure belongs to the B1 MBL group, while hydrophobic and van der Waals interactions form the dimer. The dimer form of NDM-1 can exist in both membrane-bound and purified states, which has been hypothesized to contribute to the unique resistance mechanisms [3]. NDM-1 can catalyze the hydrolysis and substrate selectivity in a wide range of β-lactam antibiotics, except for monobactam. In previous studies, l-captopril, a medicine used in hypertension treatment, showed a promising capacity to inhibit NDM-1. Furthermore, captopril derivatives were also experimented to investigate the potential to become NDM-1 inhibitors [4,5]. It has been proven that d-captopril could strongly inhibit some bacteria strains possessing the B1 MBL subfamily, such as NDM-1, IMP-1, and BcII. The pharmacokinetic profile of captopril and thiorphan reveals that in vitro inhibitory concentrations of MBLs can be attained by the usual dose [6,7]. However, the usual dose could not recover the imipenem sensitivity [8]. Captopril has not been approved to treat infections caused by bacteria excreting the NDM-1 enzyme so that the selective pressure on the evolution of the existing mutations has not been highly exerted.

Until now, NDM-1 has 40 variant mutants [9], with most point mutations occurring outside the active site and are unrelated to the increase in hydrolytic activity of this enzyme. As a result, catalytic hydrolysis is not the primary mechanism of bacteria that produce NDM-1. However, increasing the stability and affinity of zinc ions could help bacteria survive in zinc-scarce environments. The studies of Alejandro J. Vila et al. and Walter Fast et al. have shown that Met154Leu, a frequent mutant, could facilitate the hydrolysis process as well as contribute to the affinity of zinc (II) ion with N-amide in the b-lactam ring to increase ß-lactam resistance in the low concentration of zinc (II) [10,11]. There has been no study evaluating the causality in the hydrolysis capacity of NDM-1 mutants toward inhibitors in the active site. Therefore, this study aims to identify which mutants could increase or decrease the effectiveness of antibiotics toward bacteria. Specifically, two carbapenems were used to test which mutants in the NDM-1 protein could reduce the hydrolysis capacity of the enzyme toward the antibiotics. The wild-type NDM-1 enzymes of *Klebsiella pneumoniae* and *Escherichia coli* with various crystal structures (PDB ID: 5ZJ2, 4EXS, 4U4L, 5A5Z) from the protein data bank were re-docked to identify which protein structure was most suitable for further research through the interaction between the ligand and crucial amino acids [3]. The one-point substitution mutations were created based on the PAM-1 matrix by Sybyl-X 2.0 software in the interacted positions. After that, the wild-type and mutant proteins were docked with five ligands, namely d-captopril, l-captopril, thiorphan, imipenem, and meropenem, to find out the changes in docking scores and protein-ligand interaction. Finally, the complexes with increased docking scores of more than 50% were then molecular dynamically simulated and estimated binding free energy by the GROMACS software.

## 2. Results

### 2.1. NDM-1 Wild-Type Protein Structure

Among different X-ray structures from the protein data bank (http://www.rcsb.org, accessed on 1 September 2022) with resolution under 3 Å and without any missing residues in the binding pocket, the structure with PDB id 5ZJ2 was chosen because of the lowest RMSD value after re-docking with co-crystallized ligand in three conformations. Regarding the binding mode between NDM-1 and d-captopril analyzed by MOE software, the important residues include Met67, Phe70, Val73, Trp93, His120, His122, Asp124, His189, Cys208, Gly219, Asn220, and His250, which would be the mutative positions according to PAM-1 matrix. The 35 different types of highly frequent mutation of NDM-1 are described in Table 1.

### 2.2. Binding Affinity of NDM-1 Wild-Type and Mutative Proteins

Table 2 shows the docking scores of d-captopril, l-captopril, thiorphan, imipenem, and meropenem in the complex with the NDM-1 wild-type, ranging between –20.75 kJ.mol^−1^ and –30.78 kJ.mol^−1^. The interaction between these ligands with the proteins also includes crucial residues in the active sites (Table S1).

**Figure 2:**
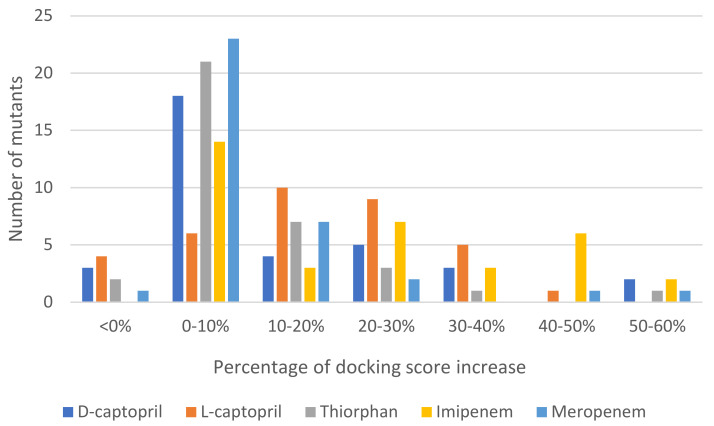
Statistics of 35 mutations of NDM-1 docked to each ligand and the percentage of docking score increase.

Regarding mutative proteins, d-captopril/H122R, d-captopril/H189Q, imipenem/H122Q, imipenem/D124A, thiorphan/D124A, and meropenem/H122R complexes are those having highly increased docking score, with the percentage of 57.67%, 59.89%, 55.98%, 54.43%, 50.35%, and 57.67%, respectively (Table 3). In general, most of the structures had an increase in docking score under 10% (Figure 2). The docking scores of ligands with NDM-1 mutant structures are shown in the supporting information (Tables S2–S6). The mutations on His122, His189, Asp124, His250, and Asn220 residues, which are related to two zinc ions, would play a vital role in enhancing the antagonism between NDM-1 protein and its inhibitors. The H122R mutation results in the decrease in docking scores (23.78%) when binding to thiorphan (Table S7). Therefore, the H122R mutation is predicted to be a positive agent in the fight against antibiotic resistance.

#### 2.2.1. D-Captopril Ligand

**Figure 3:**
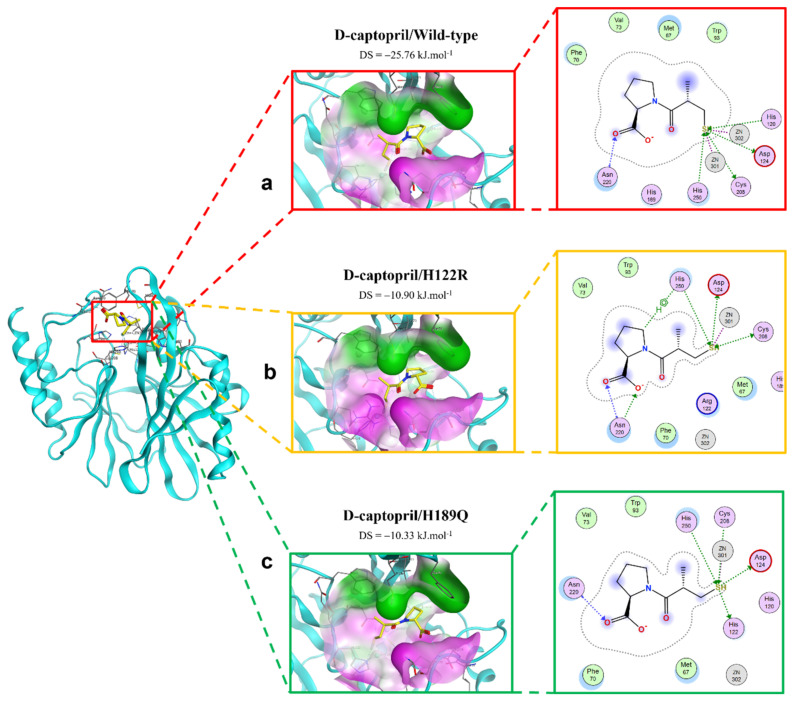
The binding site of d-captopril with NDM-1 wild-type and mutant proteins. (**a**) Wild type. (**b**) H122R. (**c**) H189Q.

Figure 3 shows the binding modes of d-captopril with NDM-1 wild-type and mutative proteins. H122R and H189Q mutations decrease the number of interactions and prohibit the connection between the ligand and Zn2, which is responsible for the stabilization of the nitrocefin-derived reaction intermediate. In addition, d-captopril interacted with H250 residue via hydrogen bonds in the d-captopril/H122R instead of H120 residue that is coordinated with Zn1, while d-captopril formed interaction with H122 residue but did not interact with H120 residue in the d-captopril/H189Q complex.

#### 2.2.2. Imipenem Ligand

**Figure 4:**
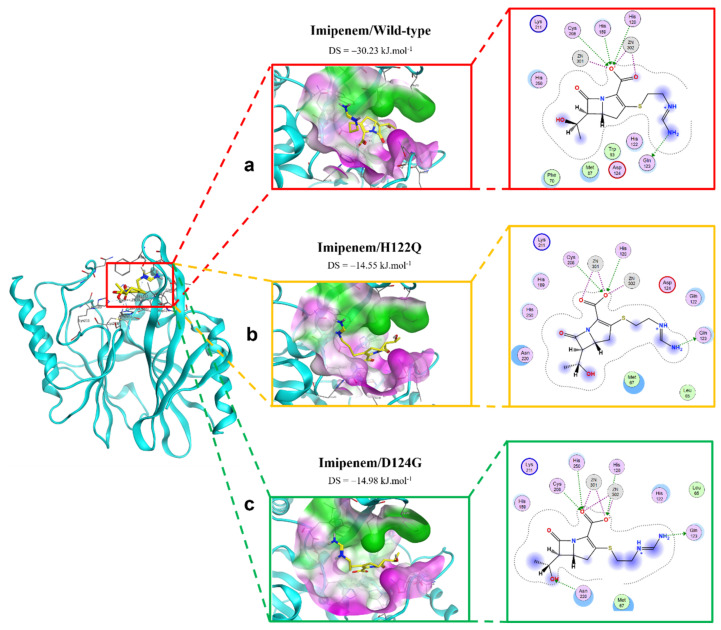
The binding site of imipenem with NDM-1 wild-type and mutant proteins. (**a**) Wild type. (**b**) H122Q. (**c**) D124G.

The interactions between imipenem and NDM-1 wild-type and mutative proteins are shown in Figure 4. The H122Q and D124A mutations reduce the binding affinity between imipenem and NDM-1 protein. Although the two mutant proteins still formed interaction with imipenem via Zn1 and Zn2 at the COO- group, there are fewer contacts with other residues in the binding pocket (Figure 4). This might be the reason why the two mutations decrease binding affinity toward imipenem.

#### 2.2.3. Meropenem Ligand

**Figure 5:**
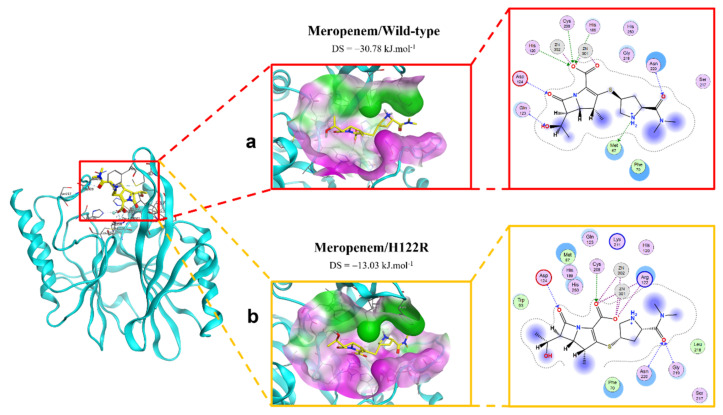
The binding site of meropenem with NDM-1 wild-type and mutant proteins. (**a**) Wild type. (**b**) H122R.

The binding modes of meropenem toward NDM-1 wild type and H122R proteins are shown in Figure 5. The number of interactions of the mutative complex was lower than the wild-type, with the reduction of hydrogen bonds at hydroxyl radical, which might highly affect the binding affinity between the ligand and protein.

#### 2.2.4. Thiorphan Ligand

**Figure 6:**
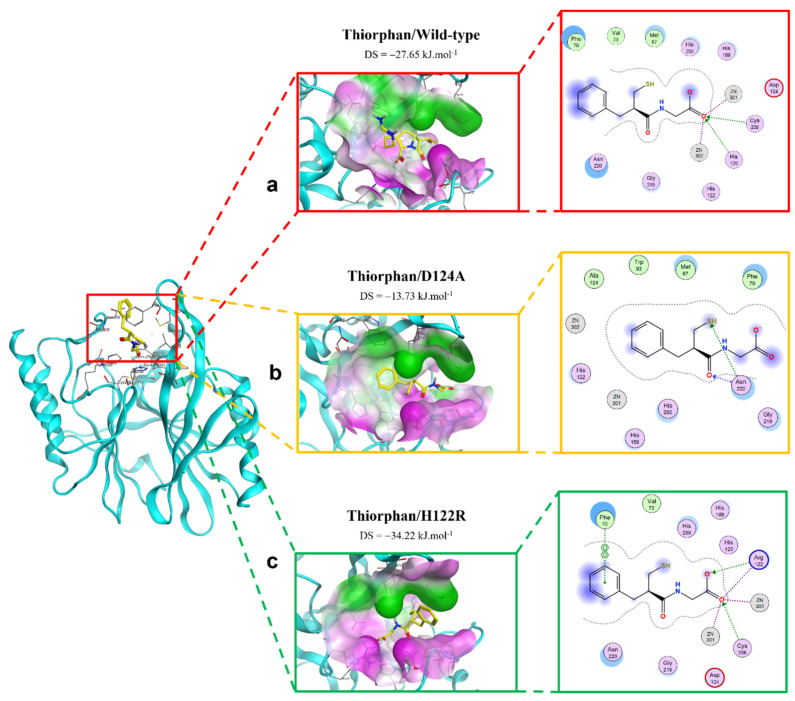
The binding site of thiorphan with NDM-1 wild-type and mutant proteins. (**a**) Wild type. (**b**) D124A. (**c**) H122R.

The extinction of ionic interactions between di-zincs (Zn1 and Zn2) and -COO^−^ in the complex of thiorphan and D124A mutation would be the reason why the binding affinity of thiorphan toward the mutative proteins significantly decreased (Figure 6). In addition, thiorphan could not form interactions with two residues related to Zn, namely His120 and Cys208. However, H122R could enhance the binding affinity of thiorphan toward NDM-1 protein. Thiorphan could form an additional hydrogen bond with the mutative residue Arg122 and become well connected with Cys208, a Zn2-coordinated amino acid (Figure 6). Therefore, the thiorphan/H122R complex is predicted to be a strong binding structure.

### 2.3. Molecular Dynamics Simulation

#### 2.3.1. The Stability of Complexes over Simulation Time

**Figure 7:**
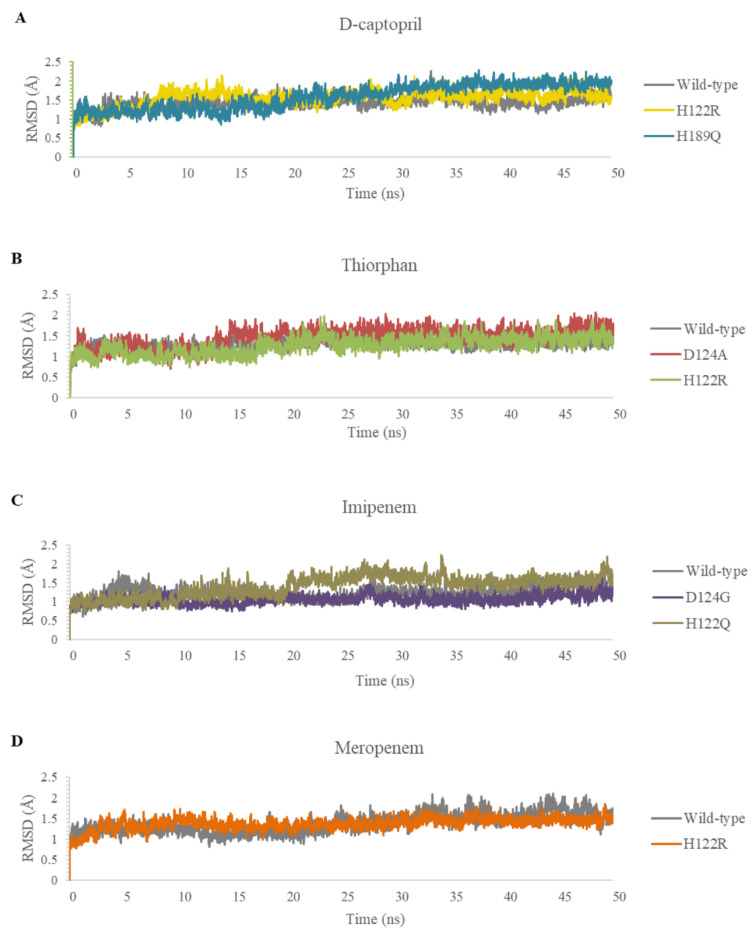
Carbon backbone RMSD profiles of the mutants and the wild type in complex with 4 ligands during MD simulations. (**A**) D-captopril. (**B**) Thiorphan. (**C**) Imipenem. (**D**) Meropenem.

In this study, the five complexes with a docking score increase more of than 50% were molecular dynamically simulated. As a result, the surveyed structure fluctuated stably around 0.7 and 2.0 Å, with the most stable structures witnessed in the wild-type complexes (Figure 7). In the last 10 ns, while other complexes continue to fluctuate sustainably, the d-captopril/H189Q complex recorded the RMSD values ranging between 2.0 and 2.2 Å (Figure 7A). During the MD simulation process, the RMSD values of H189Q and H122Q display a wider range than other mutants. Specifically, the RMSD of the d-captopril/H189Q complex increases gradually from 1.5 Å in 20 ns to reach up to 2.2 Å in 37 ns (Figure 7A). Regarding the imipenem/H122Q complex, the 20–30 ns period witnesses an unstainable fluctuation of RMSD between 1.2 and 1.9 Å, before ranging more stably under 2.0 Å in the remaining time (Figure 7C). By contrast, the wild-type protein and other mutant structures have RMSD values that remain between 0.1 and 0.2 Å throughout the molecular dynamic simulation time (Figure 7).

#### 2.3.2. The Flexibility of Residues on the Binding Site

**Figure 8:**
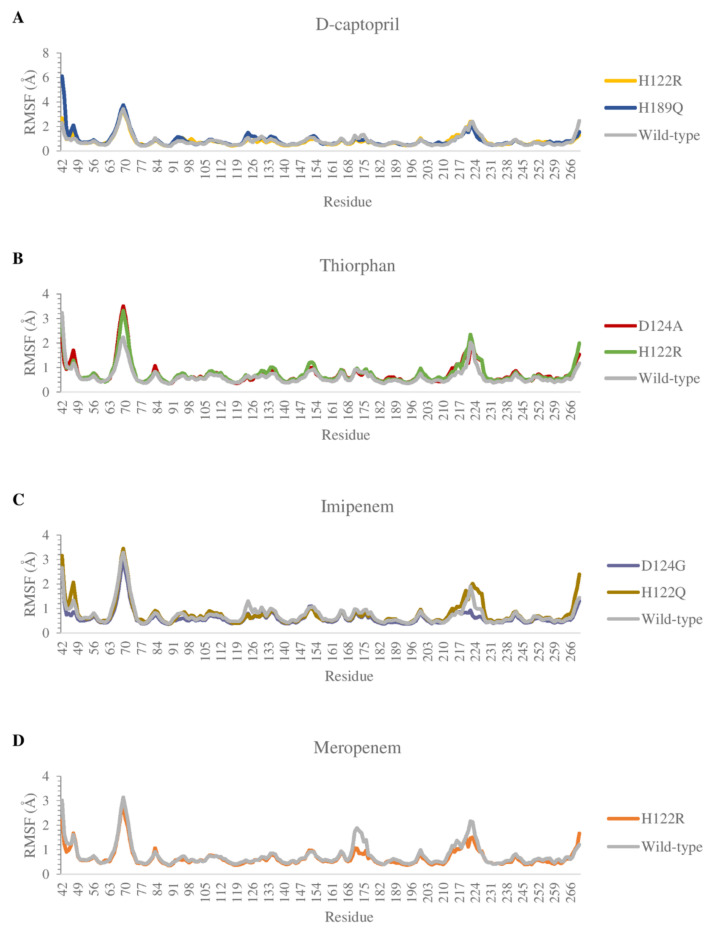
Carbon alpha RMSF values of the mutants and the wild type in complex with 4 ligands during MD simulations. (**A**) D-captopril. (**B**) Thiorphan. (**C**) Imipenem. (**D**) Meropenem.

The flexibility of residues on binding sites is shown in Figure 8. Overall, the complexes share a similar fluctuation, with most mutative proteins having RMSF values lower than wild-type complexes, especially in the mutative residues. In most of the complexes, the ligand could bind to the proteins strongly and stably, except for thiorphan/D124A and H122R, which fluctuates more unstable than thiorphan/NDM-1 wild type in the 67–71 loop (Figure 8B). Moreover, the residues between the 170 and 177 regions of the meropenem/H122R complex and 218–228 region of the imipenem/D124G complex showed minimal fluctuation compared with NDM-1 wild-type counterparts (Figure 8C,D).

#### 2.3.3. Hydrogen Bonds

##### D-Captopril

**Figure 9:**
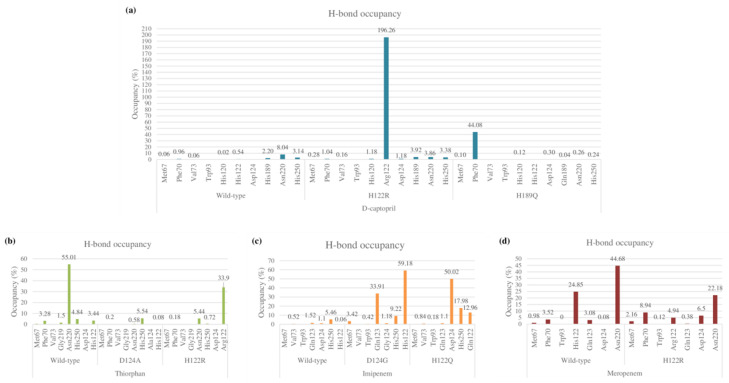
Hydrogen bond occupancy of each interacting residue in mutant and wild-type complexes during MD simulations. (**a**) D-captopril. (**b**) Thiorphan. (**c**) Imipenem. (**d**) Meropenem.

The total numbers of H-bonds forming in captopril/H122R and captopril/H189Q complexes during simulation time are about 70 bonds, which is lower when compared with 83 H-bonds of the wild-type complex (Figure S1). During the MD process, the number of hydrogen bonds between d-captopril and NDM wild-type protein in each frame varies from 0 to 8 bonds, compared to 2–10 H-bonds with thicker density recorded in d-captopril/H122R complex. The frequency of H-bonds between d-captopril and H122R protein decreases gradually during the simulation process. Regarding H189Q, although the maximum H-bonds in each frame is 16 in 3000 ps, the H-bond formation is not regular (Figure S2). The crucial residues Met67, Val73, Trp93, His120, Asp124, His189, and His250 in the three complexes gain low occupancies, with just under 3%. A noticeable change could be seen clearly in the H122R mutation when the mutated residue Arg122 could form hydrogen bonds with a higher frequency of 196.26%. In addition, the H-bonds frequency of the Phe70 residue in the H189Q mutant is significantly higher than the wild type, with 44.08% and 0.96%, respectively (Figure 9a).

##### Thiorphan

As compared to 62 hydrogen bonds found in the thiorphan/NDM-1 wild-type structure, the complex of thiorphan with D124A mutant obtains a higher number of 89 H-bonds while the thiorphan/H122R complex could form only 47 H-bonds (Figure S1). Thiorphan could form hydrogen bonds constantly with NDM-1 wild-type protein with 4–14 H-bonds per frame. By contrast, the H-bond interaction between thiorphan and H122R is unstable during the process, with the highest number of H-bonds being 9 in 4500 ps, while the figure for D124A witnessed an unusual dramatical decrease from 3250 ps to 3750 ps (Figure S2). Regarding the occupancies of hydrogen bonds of crucial amino acids, the residues Met67, Phe70, Val73, Gly219, and Asn220 in the mutant complexes formed H-bonds with a lower percentage than the wild-type structure. Especially, the frequency of H-bonds interacting with Asn220 in the wild-type structure is much higher than the D124A and H122R complexes, with 55.01% compared to 0.58% and 5.54%. However, His122 replaced by Arg122 in H122R would significantly increase the H-bonds frequency from 3.44% to 33.9%, contributing to the stronger binding ability between thiorphan and H122R protein (Figure 9b).

##### Imipenem

The imipenem ligand could form about 60 hydrogen bonds when interacting with D124G and H122Q mutant proteins during the MD simulation, which is slightly more than half of the interaction with wild-type structure (Figure S1). The H-bonds frequency in Met67, Val73, Trp93, His250, and Gln123 residues are both low in wild-type and mutant complexes. However, there is a noticeable increase in hydrogen bond occupancies in the Gln123 residue of the D124G complex from 1.52% to 33.91% and the alternative amino acid Gly124 forms H-bonds with similar frequency to the former residue. Regarding H122Q, the residues Gln122 and H250 could gain the occupancy of H-bonds higher than the wild type, with 12.96% and 17.98%, respectively, compared to 0.06% and 5.46% of the wild type (Figure 9c). The number of H-bonds in each frame of the imipenem/wild-type complex initially is deficient, but it then increases gradually to reach a peak at 20 H-bonds in 4250 ps. Meanwhile, the imipenem/D124 complex could form hydrogen bonds with a high density of 5–15 bonds during the process, which is the same as the H122Q mutant but more constantly (Figure S2).

##### Meropenem

The total number of hydrogen bonds formed during MD simulation in the meropenem/H122R complex is 49 H-bonds, lower than 78 H-bonds of the meropenem/wild-type structure (Figure S1). The number of H-bonds in every conformation, as well as the density of H-bonds during simulation time, are almost similar in both the H122R mutant and the wild-type complexes with meropenem, remaining stable in a range of 2–8 H-bonds throughout the MD simulation (Figure S2). The occupancy of hydrogen bonds between meropenem and important amino acids Met67, Phe70, Trp93, and Gln123 in both wild-type and mutant proteins are very low, with a slightly higher percentage recorded in meropenem/H122R. However, it is noticeable that the mutant position Arg122 formed H-bonds with a lower frequency than Asn122, with 4.94% and 24.85%, respectively. Moreover, the H-bonds frequency at Asn220 in the meropenem/H122R complex is just over half of the wild type (Figure 9d). Therefore, the binding affinity between meropenem and NDM-1 protein would decrease when the H122R mutation occurs, prohibiting the hydrolysis process of the H122R mutant enzyme toward the meropenem antibiotic.

### 2.4. Potential H122R Mutant in 100 ns MD Simulation

#### 2.4.1. D-Captopril/H122R Complex

**Figure 10:**
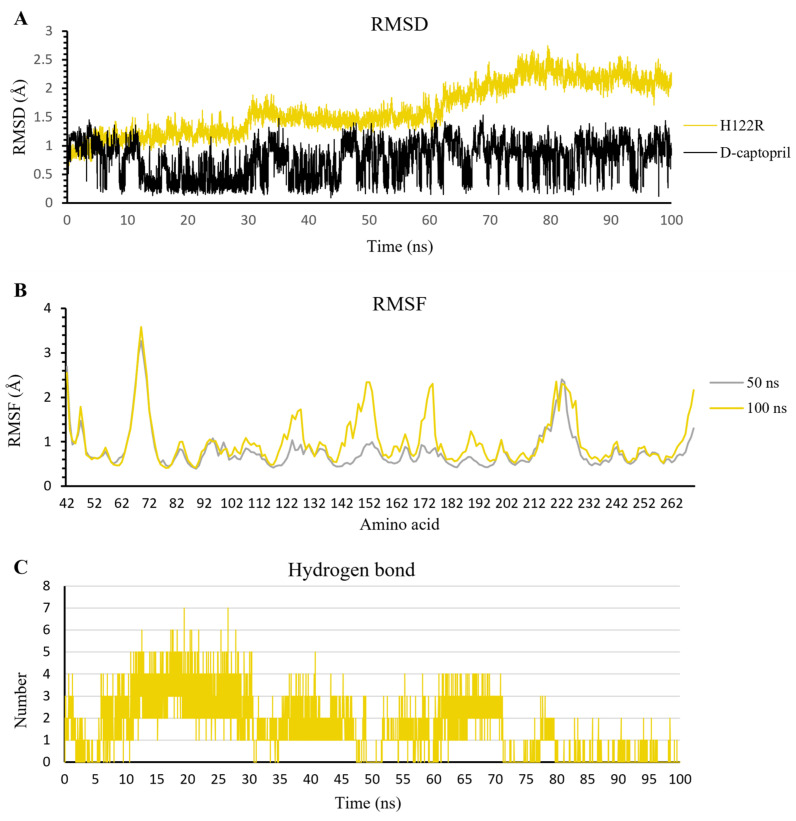
The result of d-captopril/H122R complex in 100 ns simulation time. (**A**) The RMSD value of H122R protein and d-captopril. (**B**) The RMSF value of 50 ns and 100 ns simulation time. (**C**) The average number of H-bonds formed by d-captopril with H122R mutant protein during MD simulations.

During the 100 ns extended MD simulation process, the RMSD result of protein fluctuated in a range of 1.0–3.0 Å, in which the H122R mutant remained stable in the initial 50 ns with the RMSD value under 2.0 Å, then became increased gradually to 3.0 Å for the remaining period of simulation. In contrast, the RMSD of D-captopril fluctuated in a large amplitude of 1.0 Å and was twice as high as that of the H122R mutant (Figure 10A). The RMSF result showed that the crucial residues of the 122–132 region, 142–162 region, and 182–202 region strongly fluctuated, especially the substitution at the 122nd residue (Figure 10B). This indicated that the mutation at Arg122 residue to His122 caused the unstable binding of the d-captopril/H122R complex. Regarding H-bonds, the d-captopril/H122R complex tended to form a large number of H-bonds in the initial 70 ns, around 1–7 H-bonds per frame. However, it decreased to 0–1 H-bonds per frame near the endpoint of the MD simulation (Figure 10C).

#### 2.4.2. Thiorphan/H122R Complex

**Figure 11:**
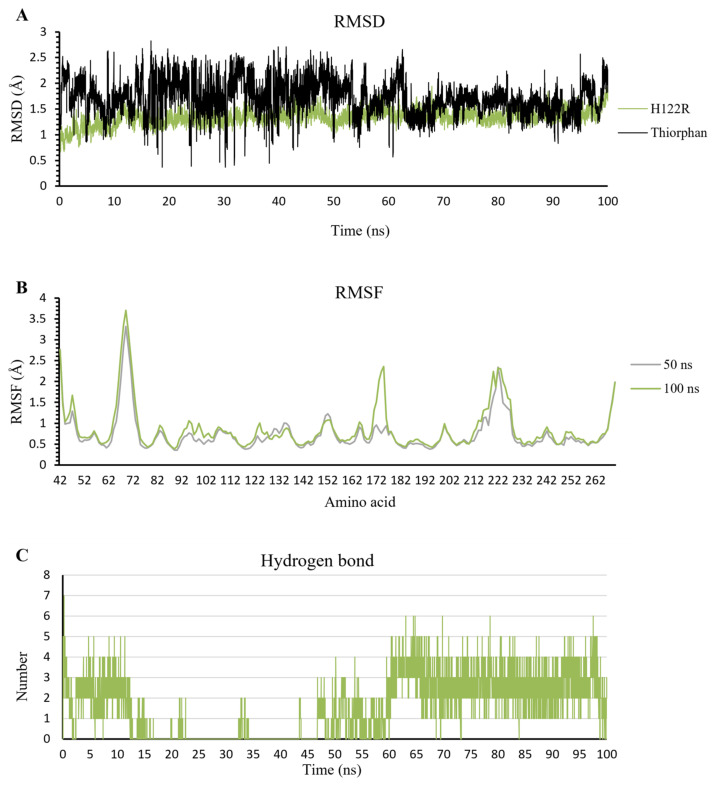
The result of thiorphan/H122R complex in 100 ns simulation time. (**A**) The RMSD value of H122R protein and thiorphan. (**B**) The RMSF value of 50 ns and 100 ns simulation time. (**C**) The average number of H-bonds formed by thiorphan with H122R mutant protein during MD simulations.

In contrast to d-captopril and meropenem, thiorphan has a more negative docking score, which has the potential to inhibit NDM-1 activation. The RMSD value of H122R protein is stable in 100 ns simulation, with the amplitude of fluctuation around 0.05 nm. Moreover, thiorphan stabilizes after 50 ns, while its oscillation in the first 50 ns is ranged from 0.5 to 2.5 Å (Figure 11A). The RMSF value in 100 ns is the same as that in 50 ns, except for the reach of a peak of 2.5 Å at 170–180 residue region (Figure 11B), but this is not the important region in the inhibitor/NDM-1 complex. Additionally, the number of hydrogen bonds has risen substantially and steadily in the last 50 ns. The H-bonds ranged from 0 to 4 bonds in the first 15 ns. There are fewer bonds formed in the next 35 ns and formed gradually around 2–3 bonds in the last 50 ns (Figure 11C). These results show the higher binding ability of thiorphan into the H122R mutant of NDM-1.

#### 2.4.3. Meropenem/H122R Complex

**Figure 12:**
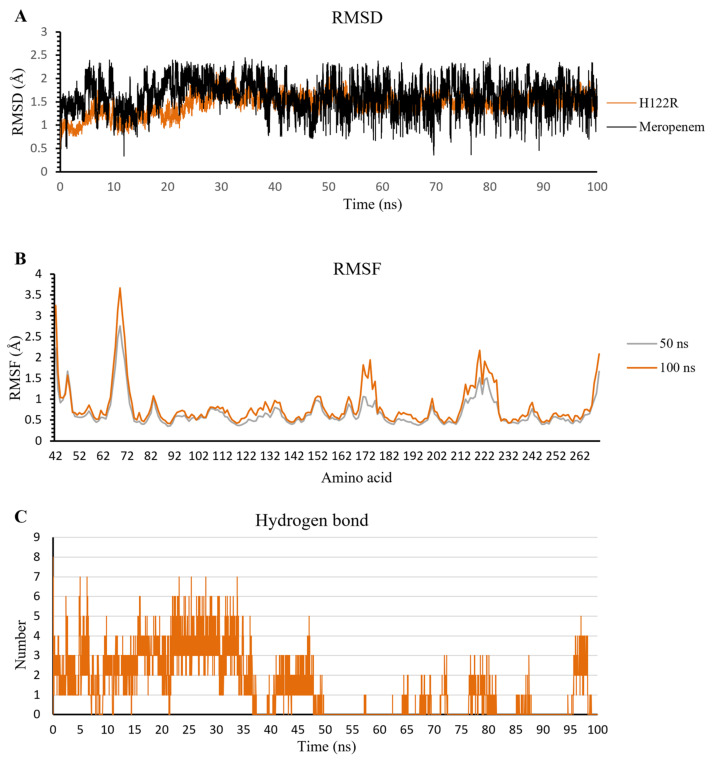
The result of meropenem/H122R complex in 100 ns simulation time. (**A**) The RMSD value of H122R protein and meropenem. (**B**) The RMSF value of 50 ns and 100 ns simulation time. (**C**) The average number of H-bonds formed by meropenem with H122R mutant protein during MD simulations.

The extended MD results in 100 ns of meropenem/H122R complex show the stable fluctuation of protein structure after the last 50 ns with an amplitude lower than 0.05 nm. In contrast to protein, meropenem has strong and continuous fluctuations in a range of 0.05–0.25 nm, and the amplitude grew up to 0.2 nm (Figure 12A). When compared to the complex at 50 ns, the RMSF value of all amino acids at 100 ns has a higher oscillation than that at 50 ns (Figure 12B). The result shows that the more simulation time of the meropenem/H122R complex, the stronger fluctuation. In addition, the hydrogen bonds decreased steadily in MD simulation, especially in the last 50 ns with a lower density of hydrogen bonds. The density of hydrogen bonds in the first 50 ns is 3–4 bonds, and that in the last 50 ns is 0–1 bonds throughout the simulation process (Figure 12C). These results support the high increase in docking score and lead to hydrolysis antibiotics of H122R.

### 2.5. Binding Free Energy

#### 2.5.1. D-Captopril and Thiorphan

**Figure 13:**
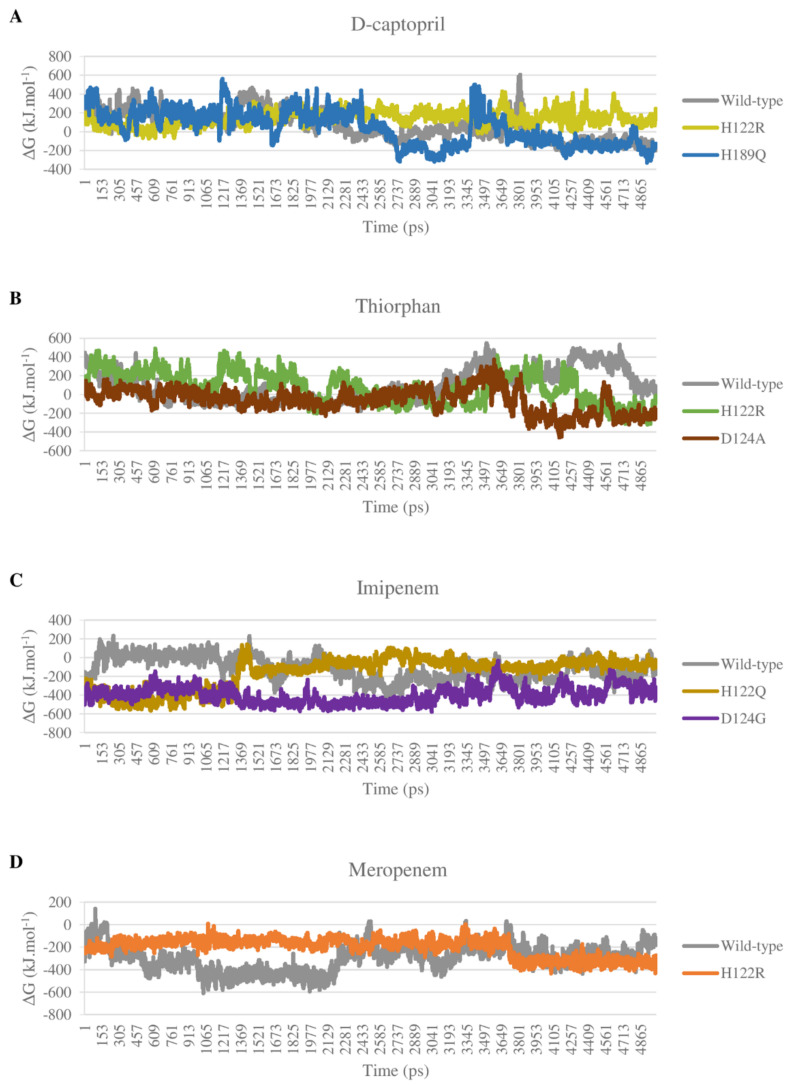
Binding energy of mutant and wild-type complexes with 4 ligands during the MD simulations. (**A**) D-captopril. (**B**) Thiorphan. (**C**) Imipenem. (**D**) Meropenem.

Most of the complexes of d-captopril and thiorphan toward NDM-1 proteins gain positive binding energies with high standard deviation, which also means the binding ability of these complexes is loose and unstable (Figure 13A,B). The result corresponds to the decrease in binding ability of the d-captopril/H122R and d-captopril/H189Q complexes with docking scores increasing more than 50%, in which van der Waals is considered as the most critical energy contributing to the binding free energy. Regarding d-captopril, the complex with H189Q remains similar to the wild type, while H122R shows a higher positive ΔG binding value of 144.404 ± 75.914 kJ·mol^−1^ compared to 65.792 ± 148.849 kJ·mol^−1^ of the wild type (Table 4). In the case of thiorphan, the binding between thiorphan and D124A mutation is more closely and stably than the thiorphan/wild-type complex, which can be seen in a negative ΔG binding value of −59.492 ± 126.395 kJ·mol^−1^ (Table 4). Although the wild-type and D124A complexes with thiorphan share similar fluctuation, the figure for D124A is significantly lower in the last 15 ns (Figure 13B). Meanwhile, the ΔG binding value of the thiorphan/H122R complex is almost the same as the wild type but fluctuates more unstably (Figure 13B).

#### 2.5.2. Imipenem and Meropenem

The binding free energy of all complexes of imipenem and meropenem is highly negative, meaning the solid and stable binding ability between the ligand and mutant protein. The electrostatic interactions play an essential role in stabilizing these complexes rather than van der Waals interactions such as d-captopril or thiorphan complexes. The imipenem complex with mutant proteins H122Q and D124G gain more negative binding energies than with NDM-1 wild-type protein, with −162.672 ± 165.792 kJ·mol^−1^, −398.543 ± 86.196 kJ·mol^−1^, and −119.611 ± 122.194 kJ·mol^−1^, respectively (Table 4). As shown in Figure 13C, the binding free energy of imipenem/D124G complex remains stable with especially low values during the process, which indicates the strong and stable binding ability of imipenem and D124G. For meropenem, the interaction with H122R protein shows higher binding energy than with the wild type with −200.489 kJ·mol^−1^ compared to −299.943 kJ·mol^−1^ (Table 4). However, H122R’s binding free energy value has a wide range of standard deviation of ± 84.547 kJ·mol^−1^. Moreover, this mutant complex’s energy also maintains stability during the process so that meropenem could bind intensely and durably to the H122R protein (Figure 13D).

## 3. Discussion

### 3.1. NDM-1 Inhibitors

Regarding the NDM-1 inhibitors, namely d-captopril and thiorphan, this study reveals that some mutants have docking scores increase while others witness a decrease in docking scores compared to the complex with NDM-1 wild-type protein. Significantly, the H122R and H189Q mutants could increase the docking scores of the d-captopril and NDM-1 complexes by more than 50%, with 57.67% and 59.89%, respectively. Besides that, the docking score of the thiorphan/D124A complex witnessed a significant increase of 50.35%, while the thiorphan/H122R complex showed the highest decrease of 23.78% in the docking score. The mutations on His122, His189, Asp124, His250, and Asn220 residues, which are related to two zinc ions, would play a vital role in enhancing the antagonism between NDM-1 protein and its inhibitors. In the study of King et al. (2011) [12], His122, His189, and Asn220 contribute to tetrahedral coordination with Zn1 and stabilize the complex, while Asp124 and His250 participate in tetrahedral coordination with Zn2. These structures were further studied through molecular dynamics simulations and binding free energy calculation.

In terms of stability, the single-point mutant has almost no effect on the complexes between NDM-1 protein and d-captopril or thiorphan during MD simulation, which can be seen in the RMSD and RMSF values. These parameters have an acceptable range during the process, with a similar fluctuation between the mutant and wild-type proteins. Regarding d-captopril, the total numbers of H-bonds in the two complexes d-captopril/H122R and d-captopril/H189Q are less than that of the d-captopril/wild-type complex, which suggests that the decline of H-bonds could be the main cause of weak interaction between d-captopril and these two mutants. However, the density of H-bonds over time in both mutant and wild-type complexes is generally unstable. In addition, at the position of some vital amino acids, the H122R and the H189Q show higher occupancy of H-bonds than the wild type, which indicates that although H122R and H189Q mutants result in significant docking scores increase, whether the interaction of these mutants with d-captopril is unsustainable remains an issue that needs to be further considered.

As a result of binding free energy, ∆the G value of the d-captopril/H122R complex increases while the figure for d-captopril/H189Q decreases compared to d-captopril/wild-type complex. Therefore, the binding free energy during the simulation time of the H189Q mutant shows that the formation of this complex is more favorable than the wild type despite the opposite docking score results. By contrast, the results from the docking process, MD simulation, and binding free energy indicate that the H122R mutant could be considered a potential mutation to reduce the binding ability between d-captopril and the NDM-1.

In the case of thiorphan, the D124A mutant witnessed an increase in the docking score of more than 50%, while the docking score of the thiorphan/H122R complex decreased most significantly among the investigated mutants. Although the occupancy of H-bonds in the D124A complex decreased significantly at Asn220 residue, the total number of H-bonds in the thiorphan/D124A interaction is more than that of the thiorphan/wild-type complex during simulation time. However, the binding free energy of the thiorphan/D124 complex is much lower than the figure for the wild-type structure. Therefore, it cannot be assumed that the significant increase in the docking score of the thiorphan/D124A would correspond with the weak binding affinity between the protein and ligand.

The interaction between H122R and thiorphan witnessed the most significant decrease in the docking score (Δ docking score = −23.78%). Despite forming fewer hydrogen bonds with lower density, the thiorphan/H122R complex gained a higher percentage of H-bonds occupancy at residue Arg122 than the previous His122 in the wild-type protein. Moreover, this mutant complex also has a lower ΔG value of 76.083 ± 164.236 kJ·mol^−1^ than 87.838 ± 167.206 kJ·mol^−1^ of the wild-type complex. With the results mentioned above, the H122R could be considered a potential mutant that promotes the binding affinity between NDM-1 protein and its inhibitor thiorphan.

### 3.2. Antibiotic Ligands

Regarding the activity on hydrolysis of NDM-1 protein toward antibiotics, most of the complexes between imipenem and meropenem with the NDM-1 mutants have higher docking scores than the wild type. There are two mutants with docking scores of imipenem increasing by more than 50%, namely D124G and H122Q. During the MD process, the two mutant complexes and the wild-type complex fluctuate stably, which can be seen in RMSD and RMSF values within the regulatory threshold. Moreover, the number of H-bonds in the mutant complexes is lower than in the wild type, with unstable H-bond frequency during the simulation. However, the two mutant proteins gain a higher frequency of H-bonds formation with imipenem at interacting residues. In the meropenem case, the H122R protein is the only mutant that could increase the docking score by more than 50% (with 57.68%). The MD simulation results of the meropenem/H122R complex reveal that this structure has similar RMSD and RMSF values as the wild type. In addition, the number of H-bonds in this complex is lower, with a smaller percentage of H-bonds occupancy recorded at Asn220 residue.

Although the above results indicated that the surveyed mutant proteins could reduce the binding ability between antibiotics toward NDM-1 protein, the binding free energy of these complexes showed a surprising opposite outcome. ∆G values of mutant complexes are significantly decreased. Different energy levels of the complex could explain the inconsistent result of binding free energy and docking score. While the ligand was prepared as an energy-minimized confirmation before docking, the complex between ligand and protein has varying free energy levels during simulation. Thus, the docking result might not entirely reflect the correlation with changes in the binding free energy of the system during the MD simulation process. In brief, the 122 and 124 residue positions could be considered most affecting the binding affinity between the NDM-1 protein and antibiotics, particularly imipenem and meropenem. However, it cannot be accurately concluded until other further in vitro and in vivo experiments.

A recent study has revealed that substitutions A233V and E152K significantly increase protein stability in the periplasm. At the same time, M154L mutation enhances the Zn(II) binding affinity leading to an increase in the hydrolyzing ability of NDM variants to antibiotics and thus causing clinically significant β-lactam resistance [10]. Ali et al. have created novel mutations near NDM-1 active sites in silico, comprising N193A, S217A, G219A, and T262A, then conducted in vitro experiments with β-lactam antibiotics on E. coli to study the role of conserved residues of NDM-1 protein. The in vitro assays showed that the IC50 values and the minimum inhibitory concentrations (MICs) of ampicillin, imipenem, meropenem, cefotaxime, cefoxitin, and ceftazidime for these mutants were decreased by about 2- to 6-fold, in comparison with the wild-type NDM-1, which means that these mutants showed a decreased resistance to these β-lactam antibiotics. These studies indicate that mutations close to NDM-1 active sites impact the capacity of hydrolyzing antibiotics and antibiotic resistance of NDM-1 protein in practice [13,14].

## 4. Materials and Methods

### 4.1. Data Collection

The structures of natural NDM-1 enzymes of two strains, *Klebsiella pneumoniae* and *Escherichia coli*, were collected from the Protein Data Bank (http://www.rcsb.org, accessed on 1 September 2022). The criteria of selected structures included: the co-crystallized ligand of protein must have a structure similar to captopril (1), the deficient amino acids were not located in the protein-ligand binding site (2), and the resolution of protein structure should be less than 3 Å (less than 2 Å is more appropriate) (3) [15].

### 4.2. Re-Docking to Find the Most Suitable Structure

The re-docking process was performed by Sybyl-X 2.0 and LeadIT 2.1.8 software, with three steps, namely protein preparation, ligand preparation, and re-docking [16,17]. In the protein preparation step, unrelated atoms were eliminated as well as some minus mistakes were corrected by LeadIT. Simultaneously, the ligand was also prepared in three types, including a co-crystallized ligand separated from the protein with its remaining confirmation, a co-crystallized ligand separated from the protein with its minimized energy conformation, and a co-crystallized ligand retrieved from the ZINC 5 database with its most stable conformation (ready-to-dock). The FlexX tool in the LeadIT software was used to dock the three ligands respectively into NDM-1 proteins. The RMSD value (root-mean-square deviation) and docking score were used to evaluate docking models in order to define the most suitable NDM-1 wild-type protein structure for the following steps.

### 4.3. Creating In Silico Mutations

The amino acids that interacted directly with the captopril ligand were determined via the Poseview tool in LeadIT, which would be the position of NDM-1 in silico alternative mutations [18]. The interacted amino acids were replaced by a high-rate one-point mutation (only one replaced amino acid in a codon [19]) based on the PAM-1 matrix. Point accepted mutation (PAM) matrix is a dataset of point mutations by natural selection that replace one amino acid with another [20]. The PAM-1 matrix corresponds to 1 mutation occurring on 100 amino acids, i.e., the probability of 1 amino acid being replaced by 1 amino acid [21]. The Sybyl-X 2.0 software created mutant proteins in a stable conformation based on Protein Composition Tool. After creating mutation, the new amino acid sequence was energy-minimized, aiming to reach a stable conformation via Minimize Edited Sequence Tool, in which the Conjugate gradient method was selected while the maximum number of iterations was set at 10,000. The energy minimization process stopped when energy changes between conformations were no more than 0.005 kcal·mol^−1^. Then, the mutant protein structure was saved in PDB format for further in silico assays.

### 4.4. Molecular Docking

In this study, the FlexX tool in the LeadIT 2.1.8 software was used to dock D-captopril, L-captopril, thiorphan, imipenem, and meropenem ligands into NDM-1 mutants, with the parameters set as follows: 1000 maximum number of solutions per iteration, 200 maximum number of solutions per fragmentation, and keep all the number of poses. Each “pose” conformation was screened manually based on the critical interactions between amino acids and ligands. The docking results of the ligands to the NDM-1 wild type and NDM-1 mutants were compared to define the effect of each mutant protein. The docking score of every complex was recorded to calculate the percentage of the changes by Formula (1), in which the lower docking score indicates stronger binding affinity while the mutant’s increased docking score might induce bacterial resistance. In addition, the number of amino acids in the complex’s interaction and hydrogen bond amount and length were also evaluated to explain the changes in docking scores [18].

(1) $$A=\frac{{\mathrm{Score}}_{\mathrm{natural}}-{\;\mathrm{Score}}_{\mathrm{mutant}}}{{\mathrm{Score}}_{\mathrm{natural}}}\times 100\%$$

As a result, if the A value is positive, the docking score of captopril with the NDM-1 mutant is lower than the NDM-1 wild type, which also means the binding ability between protein and ligand was decreased. Regarding the two antibiotics inactivated by NDM-1, namely imipenem and meropenem, the lower binding ability correlated with the decrease in hydrolysis capacity of the NDM-1 enzyme. By contrast, the percentage is negative when the docking score of captopril with the NDM-1 mutant is higher than the NDM-1 wild type, which could lead to more sensitive inhibitors (captopril and thiorphan) toward mutant proteins. Once the binding ability between the antibiotic and the NDM-1 protein increases, the drug is more readily hydrolyzed by this enzyme and becomes more resistant, such as imipenem and meropenem.

### 4.5. Molecular Dynamics Simulation

The complexes whose docking scores increased more than 50% were simulated molecular dynamics by Gromacs 2020.4. The topology of the protein was generated in the CHARMM27 force field. The ligand topology was built in CHARMM27 by the Swissparam tool at http://www.swissparam.ch, accessed on 1 September 2022 [22]. Two topology files of protein and ligand were used to create the complex topology file. The complex was solvated in a dodecahedron box of the TIP3P water model with a distance from the protein center to the box edge of 1.0 nm. Na^+^ and Cl^−^ ions were added to neutralize the charge with a salt concentration of 0.15 M. The energy minimization was performed using the steepest descent algorithm. The system proceeded with NVT equilibration to the 300 K temperature at 100 ps and NPT equilibration to the 1 bar pressure at 100 ps. After reaching equilibrium, the system was stimulated within 50 ns with time step 10 ps.

In order to evaluate the stability of apo-proteins, ligands, or protein-ligand complexes, the RMSD value was calculated by Formula (2), in which $r_{i}$(t) is the position of atom (i) at time t, and N is the number of atoms. The complex is considered stable when the RMSD < 3 Å [23]. Moreover, the fluctuation of amino acids in the protein was evaluated through the RMSF value based on Formula (3), where T is the time to average and $r_{i}^{\mathrm{ref}}$ is the reference position of atom (i). The lower RMSF value indicates that the amino acid has limited fluctuation during the simulation compared to the original site [24]. The amino acid is unstable when RMSF > 2 Å [25]. Finally, this study also calculated any hydrogen bond, one of the vital kinds of bonds in ligand bonding, in the complex that appears over simulation time and any amino acids that appear in hydrogen bonds [26] by VMD 1.9.3 software.

(2) $$\mathrm{RMSD}\left(t\right)=\sqrt{\frac{1}{M}\overset{N}{\underset{i=1}{\sum }}m_{i}{\left|r_{i}\left(t\right)-r_{i}^{\mathrm{ref}}\right|}^{2}\;}$$

(3) $${\mathrm{RMSF}}_{i}=\sqrt{\frac{1}{T}\overset{T}{\underset{t=1}{\sum }}{\left|r_{i}\left(t\right)-r_{i}^{\mathrm{ref}}\right|}^{2}}$$

Hydrogen bonds are non-covalent bonds that are formed due to an atom donating a proton to an electronegative atom [27]. H-bonds are generally considered a crucial factor that promotes protein-ligand binding despite their weak interaction since many H-bonds could form a strong interaction network. In this study, the VMD software was used to visualize H-bonds between ligand and protein in a simulation trajectory by setting two requirements, including donor-acceptor distance ≤ 3.2 Å (1) and donor-hydrogen-acceptor angle ≥ 150° (2). The evaluated values include the total number of H-bonds, the variation of the number of H-bonds over time, and the frequency of H-bonds at the crucial residues.

### 4.6. Binding Free Energy Calculation

The binding free energy was calculated based on MM-PBSA methods (molecular mechanics Poisson–Boltzmann surface area) to estimate protein-ligand binding affinity during the simulation. The g_mmpbsa tool in Gromacs [28] was used to calculate binding free energy throughout Formula (4), in which G_complex_ is the protein-ligand complex energy, G_protein_ is the total free energy of apo-protein, and Gligand is the total free energy of ligand.

(4) $$\Delta G_{\mathrm{binding}}=G_{\mathrm{complex}}-\left(G_{\mathrm{protein}}+G_{\mathrm{ligand}}\right)$$

The free energy of each system is calculated by Formula (5). However, the entropy contribution to the system is usually meager and difficult to calculate precisely. Moreover, the single trajectory approach, such as complex, often does not consider any binding-induced structural changes. Therefore, with an aim to compare the relative binding free energy of complexes, in which the entropy value is often omitted [29]. The values of ∆G_polar_, ∆G_nonpolar_, ∆E_MM_, ∆E_van der Waals_, and ∆E_electrostatic_ are calculated by Formulas (6) and (7).

(5) $$G_{x}=\langle {\;E}_{\mathrm{MM}}\rangle -\mathrm{TS}+\langle G_{\mathrm{solvation}}\rangle$$

(6) $$E_{\mathrm{MM}}=E_{\mathrm{bonded}}+E_{\mathrm{nonbonded}}=E_{\mathrm{bonded}}+\left(E_{\mathrm{vdW}}+E_{\mathrm{elec}}\right)$$

(7) $$G_{\mathrm{solvation}}=G_{\mathrm{polar}}+G_{\mathrm{nonpolar}}$$

## 5. Conclusions

Recently, the spread of bacteria expressing the NDM-1 hydrolysis enzyme has become a significant concern since these bacteria have been resistant to both carbapenems and third-generation cephalosporins, which used to be sensitive to other multidrug-resistant bacteria [30]. The resistance to carbapenem antibiotics of bacteria, especially for Enterobacteriaceae strains, including *Klebsiella*, *E. coli*, *Serratia*, and *Proteus*, has been alerted urgently by WHO. With the intention of evaluating the ability of antibiotic resistance as well as the capacity of hydrolyzing antibiotics of NDM-1 mutants, the study was conducted to investigate the binding ability of the alternative one-point mutants toward inhibitors, namely l-captopril, d-captopril, and thiorphan; as well as the capacity of hydrolyzing antibiotics namely imipenem and meropenem. The study selected seven mutants with docking scores increasing by more than 50% compared to the wild type to conduct MD simulation and calculate binding free energy, including H122R and H189Q with d-captopril, H122R and D124A with thiorphan, H122Q and D124G with imipenem, and H122R with meropenem. The MD simulation outcome shows that the seven mutant complexes are stable during 50 ns simulation time. In addition, this study found that the H122R mutant has the resistance ability to d-captopril but increases the sensitivity to thiorphan. Besides that, although H189Q and D124A mutants might highly impact the binding ability of d-captopril and thiorphan toward NDM-1 protein, the decline of these interactions could not be declared. Regarding imipenem and meropenem antibiotics, there is a more than 50% increase in the docking scores of H122Q, D124G, and H122R complexes. However, the conflict of binding free energy values would lead to difficulty in exactly concluding the capacity to hydrolyze these antibiotics by NDM-1 mutant hydrolysis enzyme. After extending the MD simulation process of H122R to 100 ns, the results showed that the H122R mutant tended to decrease the stable binding ability of inhibitors, including d-captopril and meropenem, while increasing the binding affinity with an antibiotic such as thiorphan. These results supported that the H122R mutant of NDM-1 increases the resistance ability to d-captopril and meropenem but enhances the sensitivity to thiorphan.

In conclusion, this in silico study reveals that the H122R mutation could potentially reduce the binding ability toward d-captopril and, thereby, might highly affect the resistance of NDM-1-expressing bacteria. However, there must be further in vitro and in vivo experiments in the future to confirm this hypothesis. Moreover, other mutant proteins with docking scores increased from 30% to 50% should also be evaluated in the following research.

## Figures and Tables

**Table 1:** High probable mutation of NDM-1 protein according to PAM-1 matrix.

Amino Acid	High Frequent Mutation of NDM-1					Number
Met67	M67L	M67V	M67I	M67K		4
Phe70	F70L	F70Y				2
Val73	V73A	V73I				2
Trp93	W93R	W93L	W93S			3
His120	H120R	H120N	H120Q			3
His122	H122R	H122N	H122Q			3
Asp124	D124A	D124N	D124E	D124G		4
His189	H189R	H189N	H189Q			3
Cys208	C208S					1
Gly219	G219A	G219S				2
Asn220	N220G	N220H	N220K	N220S	N220T	5
His250	H250R	H250N	H250Q			3
Total						35

**Table 2:** Docking score of ligands with NDM-1 protein (PDB ID: 5ZJ2).

Ligand	Structure	Docking Score (kJ·mol−1)
D-captopril	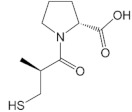	−25.76
L-captopril	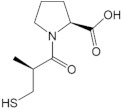	−20.75
Thiorphan	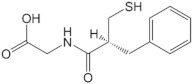	−27.65
Imipenem	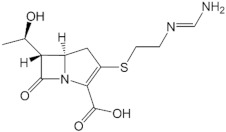	−30.23
Meropenem	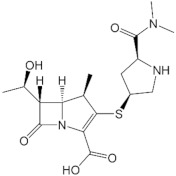	−30.78

**Table 3:** Mutants with a high percentage increase in docking score in the ligand binding assay.

Ligand	Mutant					
D-captopril	H189Q	H122R	H250N	N220G	H250R	
Percentage increase in docking score (%)	59.89	57.67	39.48	32.76	32.04	
L-captopril	N220G	H250N	H189Q	N220H	N220S	D124E
Percentage increase in docking score (%)	45.42	39.01	37.68	36.90	36.2	30.29
Thiorphan	D124A	H189Q	H122R			
Percentage increase in docking score (%)	50.35	30.69	−23.78			
Imipenem	H122Q	H122R	D124N	D124E	D124A	H122N
Percentage increase in docking score (%)	55.98	48.81	44.36	38.91	36.07	33.87
Meropenem	D124G	H189N	H189Q	H189R	G219S	
Percentage increase in docking score (%)	54.43	49.79	42.89	41.45	38.84	

**Table 4:** The results of binding free energy of mutant and wild-type complexes with 4 ligands.

Ligand	Structure	ΔE_vdW_ (kJ·mol^−1^)	ΔE_elec_ (kJ·mol^−1^)	ΔG_pol_ (kJ·mol^−1^)	ΔG_nonpol_ (kJ·mol^−1^)	ΔG_bind_ (kJ·mol^−1^)
D-captopril	Wild type	−50.970 ± 22.061	80.759 ± 191.470	43.346 ± 67.767	−7.343 ± 2.844	65.792 ± 148.849
	H122R	−42.950 ± 28.844	80.925 ± 92.689	114.204 ± 77.055	−7.774 ± 3.800	144.404 ± 75.914
	H189Q	−23.138 ± 27.542	−11.116 ± 235.609	84.664 ± 94.956	−4.140 ± 3.599	46.270 ± 183.341
Thiorphan	Wild type	−72.670 ± 16.206	51.009 ± 263.722	121.157 ± 112.454	−11.658 ± 2.345	87.838 ± 167.206
	H122R	−32.179 ± 28.586	37.285 ± 234.204	76.746 ± 101.440	−5.770 ± 4.223	76.083 ± 164.236
	D124A	−54.567 ± 26.739	−74.501 ± 212.301	77.550 ± 96.705	−7.975 ± 3.283	−59.492 ± 126.395
Imipenem	Wild type	−32.915 ± 26.014	−217.441 ± 214.214	137.187 ± 126.093	−6.442 ± 4.127	−119.611 ± 122.194
	H122Q	−56.520 ± 17.858	−331.121 ± 166.963	235.477 ± 87.403	−10.508 ± 1.873	−162.672 ± 165.792
	D124G	−38.956 ± 22.490	−745.386 ± 143.139	397.213 ± 78.758	−11.414 ± 3.015	−398.543 ± 86.196
Meropenem	Wild type	−52.850 ± 30.194	−399.750 ± 167.545	162.424 ± 85.753	−9.767 ± 4.724	−299.943 ± 116.132
	H122R	−95.839 ± 17.361	−165.440 ± 90.794	72.914 ± 40.381	−12.123 ± 1.984	−200.489 ± 84.547

## Data Availability

Not applicable.

## Supplementary Materials

The supporting information can be downloaded at: https://www.mdpi.com/article/10.3390/ijms232416083/s1.
