# Supplementary material for: The Effects of One-Point Mutation on the New Delhi Metallo Beta-Lactamase-1 Resistance toward Carbapenem Antibiotics and β-Lactamase Inhibitors: An In Silico Systematic Approach
Source: Int J Mol Sci. 2022 Dec 16;23(24):16083. doi: 10.3390/ijms232416083 (PMC9785264; doi:10.3390/ijms232416083)

SUPPORTING INFORMATION

The effects of alternative one-point mutation on the resistance of New Delhi Metallo beta-lactamase-1 towards carbapenem antibiotics and β-lactamase inhibitors: an *in silico* systematically approach

Van-Thanh Tran[1,†], Viet-Hung Tran [2,*,†], Dac-Nhan Nguyen[1], Tran-Giang-Son Do [1], Thanh-Phuong Vo [1], Thi-Thao-Nhung Nguyen [1], Phuong Nguyen Hoai Huynh [1], and Khac-Minh Thai[1,*]

[1] Faculty of Pharmacy, University of Medicine and Pharmacy at Ho Chi Minh City, Ho Chi Minh City, 700000 Vietnam

[2] Institute of Drug Quality Control Ho Chi Minh City, Ho Chi Minh City, 700000, Vietnam

* Correspondence: tran.viethung168@gmail.com (Viet-Hung Tran); thaikhacminh@ump.edu.vn; thaikhacminh@gmail.com (Khac-Minh Thai).

† These authors contributed equally to this work.

Table S1 Amino acid and atoms in binding site interacting with ligands

**Ligand**	**The interacted amino acids in the binding pocket of protein-ligand complexes**																
	**Met 67**	**Phe 70**	**Val 73**	**Trp 93**	**His 120**	**His 122**	**Gln 123**	**Asp 124**	**His 189**	**Gly 219**	**Asn 220**	**His 250**	**Zn 301**	**Zn 302**	**HOH 405**	**HOH 409**	**HOH 500**
**D-captopril**	**x**	**x**	**x**	**x**	**X**	**x**		**x**	**x**		**x**	**x**	**x**		**x**	**x**	**x**
**L-captopril**	**x**	**x**	**x**		**X**	**x**			**x**		**x**	**x**	**x**		**x**	**x**	
**Thiorphan**	**x**	**x**	**x**							**x**	**x**	**x**	**x**	**x**	**x**		**x**
**Imipenem**	**x**		**x**	**x**		**x**	**x**	**x**				**x**	**x**	**x**	**x**	**x**	**x**
**Meropenem**	**x**	**x**		**x**		**x**	**x**	**x**			**x**		**x**	**x**			**x**

Table S2 The docking scores (kJ.mol^{-1}) of **d-captopril** with NDM-1 mutant structures

D-captopril	Met67	Phe70	Val73	Trp93	His120	His122	Asp124	His189	Cys208	Gly219	Asn220	His250
**Ala/A**			-25.6				-23.5			-24.09		
**Arg/R**				-24.4	-24.29	-10.9		-24.43				-17.5
**Asn/N**					-24.46	-22.01	-22.6	-19.24				-15.59
**Asp/D**												
**Cys/C**												
**Gln/Q**					-24.46	-18.62		-10.33				-21.06
**Glu/E**							-18.51					
**Gly/G**							-22.12				-17.32	
**His/H**											-18.22	
**Ile/I**	-24.04		-25.6									
**Leu/L**	-25.06	-25.2		-24.7								
**Lys/K**	-24.31										-29.45	
**Met/M**												
**Phe/F**												
**Pro/P**												
**Ser/S**				-24.7					-25.05	-25.88	-26.53	
**Thr/T**											-18.57	
**Trp/W**												
**Tyr/Y**		-24.9										
**Val/V**	-25.06											

Note:

**Percentage of docking score increase**						
**<0% (Group 1)**	**0-10% (Group 2)**	**10-20% (Group 3)**	**20-30% (Group 4)**	**30-40% (Group 5)**	**40-50% (Group 6)**	**50-60% (Group 7)**

Table S3 The docking scores (kJ.mol^{-1}) of **l-captopril** with NDM-1 mutant structures

L-captopril	Met67	Phe70	Val73	Trp93	His120	His122	Asp124	His189	Cys208	Gly219	Asn220	His250
Ala/A			-21.25				-16.78			-17.23		
Arg/R				-17.04	-20.23	-15.06		-17.39				-18.41
Asn/N					-15.75	-19.14	-15.95	-16.94				-12.66
Asp/D												
Cys/C												
Gln/Q					-20.72	-15.64		-12.93				-17.48
Glu/E							-14.47					
Gly/G							-16.33				-11.33	
His/H											-13.10	
Ile/I	-22.22		-20.98									
Leu/L	-19.25	-16.12		-20.75								
Lys/K	-16.16										-20.91	
Met/M												
Phe/F												
Pro/P												
Ser/S				-17.33					-17.68	-18.15	-13.23	
Thr/T											-15.99	
Trp/W												
Tyr/Y		-16.10										
Val/V	-19.08											

Note:

Percentage of docking score increase						
<0% (Group 1)	0-10% (Group 2)	10-20% (Group 3)	20-30% (Group 4)	30-40% (Group 5)	40-50% (Group 6)	50-60% (Group 7)

Table S4 The docking scores (kJ.mol^{-1}) of **thiorphan** with NDM-1 mutant structures

Thiorphan	Met67	Phe70	Val73	Trp93	His120	His122	Asp124	His189	Cys208	Gly219	Asn220	His250
**Ala/A**			-22.2				-13.73			-24.31		
**Arg/R**				-21.5	-27.65	-34.22		-25.71				-30.62
**Asn/N**					-27.65	-25.15	-24.98	-25.63				-22.95
**Asp/D**												
**Cys/C**												
**Gln/Q**					-27.65	-19.55		-19.16				-21.38
**Glu/E**							-24.87					
**Gly/G**							-24.18				-24.78	
**His/H**											-25.59	
**Ile/I**	-24.62		-26.9									
**Leu/L**	-25.49	-26.4		-24.9								
**Lys/K**	-27.9										-23.71	
**Met/M**												
**Phe/F**												
**Pro/P**												
**Ser/S**				-27.6					-24.97	-26.54	-22.74	
**Thr/T**											-25.31	
**Trp/W**												
**Tyr/Y**		-26.6										
**Val/V**	-25.65											

Note:

Percentage of docking score increase						
**<0% (Group 1)**	**0-10% (Group 2)**	**10-20% (Group 3)**	**20-30% (Group 4)**	**30-40% (Group 5)**	**40-50% (Group 6)**	**50-60% (Group 7)**

Table S5 The docking scores (kJ.mol^{-1}) of **imipenem** with NDM-1 mutant structures

Imipenem	Met67	Phe70	Val73	Trp93	His120	His122	Asp124	His189	Cys208	Gly219	Asn220	His250
**Ala/A**			-28				-20.13			-27.8		
**Arg/R**				-29.2	-28.57	-16.55		-18.62				-22.77
**Asn/N**					-26.14	-20.74	-17.8	-16.28				-24.96
**Asp/D**												
**Cys/C**												
**Gln/Q**					-28.6	-14.55		-18.21				-23.63
**Glu/E**							-19.33					
**Gly/G**							-14.98				-27.54	
**His/H**											-29.39	
**Ile/I**	-22.06		-30.1									
**Leu/L**	-28.02	-28.1		-28.7								
**Lys/K**	-22.06										-25.16	
**Met/M**												
**Phe/F**												
**Pro/P**												
**Ser/S**				-28.8					-23.19	-19.35	-22.37	
**Thr/T**											-25.67	
**Trp/W**												
**Tyr/Y**		-28										
**Val/V**	-22.87											

Note:

**Percentage of docking score increase**						
**<0% (Group 1)**	**0-10% (Group 2)**	**10-20% (Group 3)**	**20-30% (Group 4)**	**30-40% (Group 5)**	**40-50% (Group 6)**	**50-60% (Group 7)**

Table S6 The docking scores (kJ.mol^{-1}) of **meropenem** with NDM-1 mutant structures

Meropenem	Met67	Phe70	Val73	Trp93	His120	His122	Asp124	His189	Cys208	Gly219	Asn220	His250
Ala/A			-30.7				-26.68			-28.16		
Arg/R				-30.1	-26.02	-13.03		-30.88				-38.58
Asn/N					-27.43	-33.18	-22.97	-30.71				-28.94
Asp/D												
Cys/C												
Gln/Q					-27.43	-24.83		-26.37				-28.95
Glu/E							-16.5					
Gly/G							-29.59				-28.37	
His/H											-29.03	
Ile/I	-29.54		-30.8									
Leu/L	-29.68	-28.3		-30.1								
Lys/K	-30.18										-25.83	
Met/M												
Phe/F												
Pro/P												
Ser/S				-30.2					-29.83	-28.79	-28.35	
Thr/T											-30.75	
Trp/W												
Tyr/Y		-24.2										
Val/V	-29.02											

Note:

Percentage of docking score increase						
<0% (Group 1)	0-10% (Group 2)	10-20% (Group 3)	20-30% (Group 4)	30-40% (Group 5)	40-50% (Group 6)	50-60% (Group 7)

Table S7 Percentage increase in docking score (%) between NDM-1 mutants and ligands

Acid amin	Mutant	Ligand				
		D-captopril	L-captopril	Thiorphan	Imipenem	Meropenem
Met67	M67L	2.69	7.23	8.32	7.89	3.58
	M67V	2.69	8.06	7.21	26.28	5.73
	M67I	2.66	-7.07	10.96	29.17	4.04
	M67K	5.6	22.13	-0.94	29.17	1.95
Phe70	F70L	2.29	22.35	4.40	7.78	7.91
	F70Y	3.18	22.44	3.85	8.06	21.27
Val73	V73A	0.47	-2.373	19.74	8.06	0.27
	V73I	0.64	-1.09	2.56	0.32	0.09
Trp93	W93R	5.34	17.89	22.07	3.57	2.32
	W93L	4.09	0.027	10.04	5.64	2.32
	W93S	4.09	16.47	0.00	5.10	1.77
His120	H120R	5.69	2.52	0.00	5.95	15.47
	H120N	5.03	24.09	0.00	14.61	10.89
	H120Q	5.03	0.15	0.00	5.85	10.89
His122	H122R	57.67	27.45	-23.78	48.81	57.68
	H122N	14.54	7.77	9.02	33.87	-7.80
	H122Q	27.7	24.62	29.30	55.98	19.34
Asp124	D124A	8.77	19.15	50.35	36.07	13.30
	D124N	12.24	23.15	9.65	44.36	25.38
	D124E	28.12	30.29	10.03	38.91	46.38
	D124Q	14.12	21.29	12.55	54.43	3.87
His189	H189R	5.15	16.22	7.01	41.45	-0.32
	H189N	25.29	18.36	7.30	49.79	0.23
	H189Q	59.89	37.68	30.69	42.89	14.33
Cys208	C208S	2.79	14.81	9.66	25.12	3.07
Gly219	G219A	6.477	16.99	12.06	8.67	8.50
	G219S	-0.47	12.54	4.01	38.84	6.47
Asn220	N220G	32.76	45.42	10.38	9.61	7.81
	N220H	29.25	36.89	7.44	3.00	5.70
	N220K	-14.36	-0.74	14.25	18.11	16.07
	N220S	-3.01	36.25	17.73	28.06	7.89
	N220T	27.89	22.93	8.46	16.29	0.09
His250	H250R	32.04	11.27	-10.77	26.64	-25.36
	H250N	39.48	39.01	17.00	18.83	5.97
	H250Q	18.22	15.79	22.68	23.56	5.95

Note:

Percentage of docking score increase						
<0% (Group 1)	0-10% (Group 2)	10-20% (Group 3)	20-30% (Group 4)	30-40% (Group 5)	40-50% (Group 6)	50-60% (Group 7)

Figure S1 The total numbers of H-bonds forming in mutant and wild type complexes

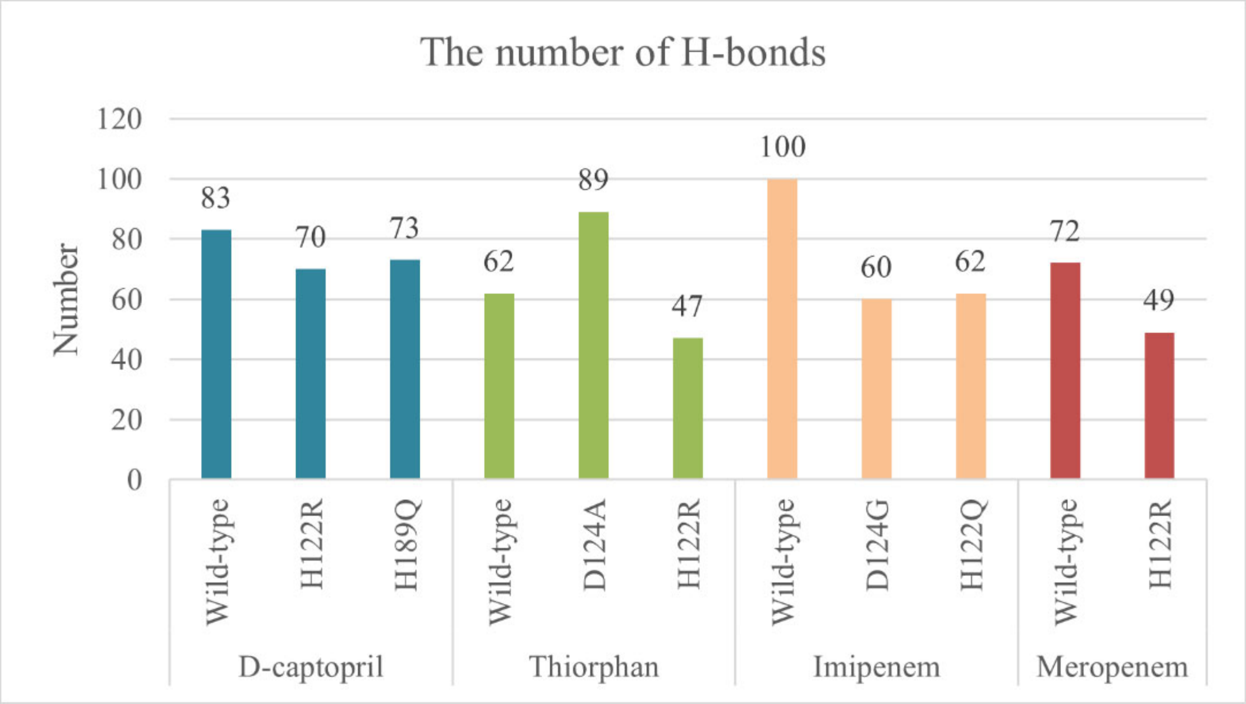

Figure S2 The average number of H-bonds formed by the ligands with mutant and wild type NDM-1 protein during MD simulations

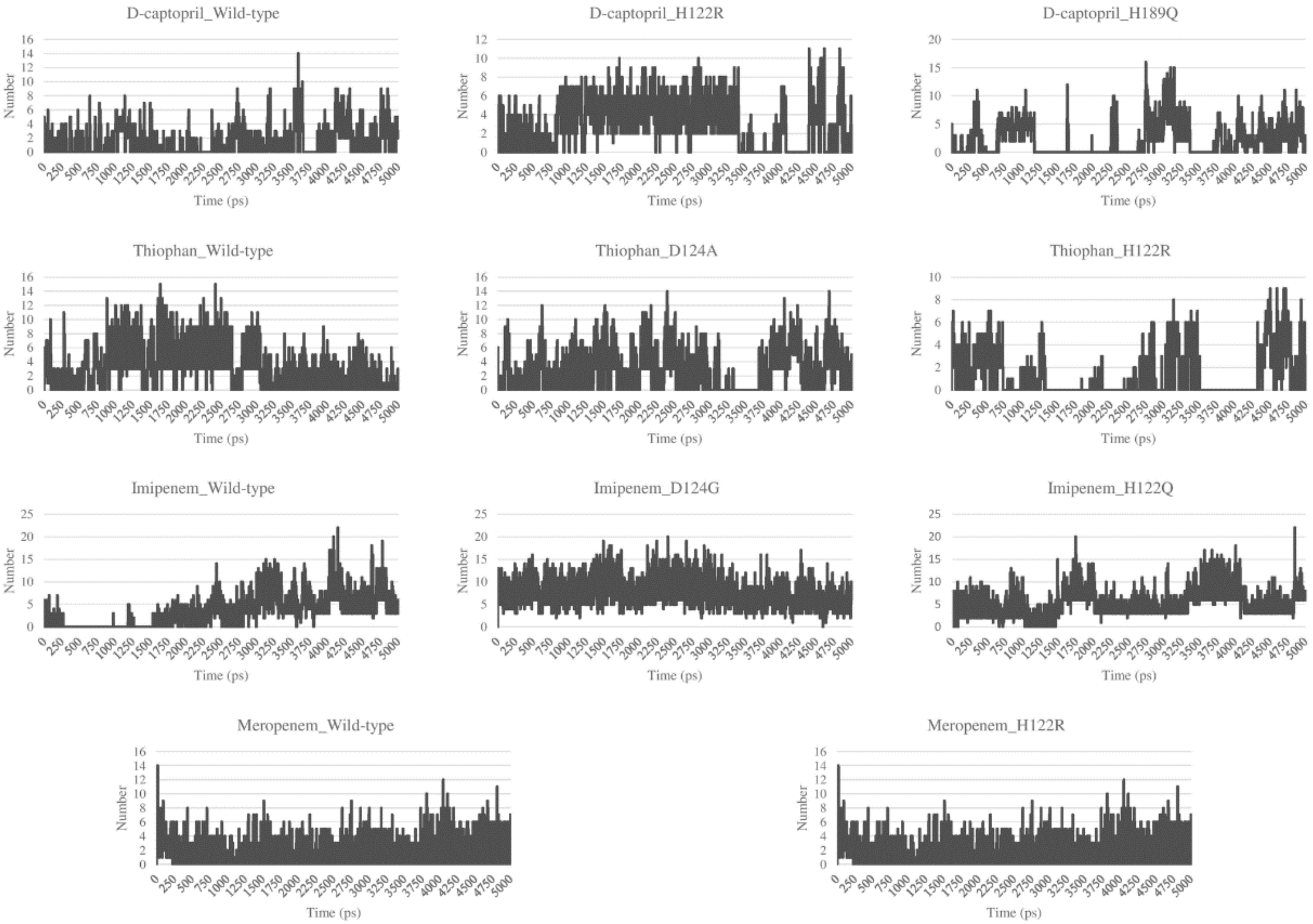

Supplement: Supplementary file 1 [file ijms-23-16083-s001.zip › ijms-2008362-supplementary.pdf]
